# Epstein–Barr Virus Infection in Cancer

**DOI:** 10.3390/cancers15184659

**Published:** 2023-09-21

**Authors:** Lucia Mundo, Lorenzo Leoncini, Rosita Accardi-Gheit

**Affiliations:** 1 Health Research Institute, School of Medicine, University of Limerick, V94 T9PX Limerick, Ireland; 2 Section of Pathology, Department of Medical Biotechnologies, University of Siena, 53100 Siena, Italy; 3 Section of Mechanisms of Carcinogenesis, International Agency for Research on Cancer, World Health Organization, 69366 Lyon, France

## 1. Introduction

EBV was the first human oncogenic virus identified. It was discovered in tumour cells isolated from Burkitt’s lymphoma tissue by Sir Anthony Epstein and Dr Yvonne Barr in 1964. Some years after its discovery, EBV was shown to be able to transform normal leukocytes into lymphoblastoid cell lines (LCLs). Since then, EBV has been found to be associated with many malignancies originating from lymphocytes or epithelial cells (Burkitt’s lymphoma, post-transplant and HIV-associated lymphomas, Hodgkin’s lymphoma, T-cell lymphoma, extra-nodal nasal-type natural killer/T-cell lymphoma, nasopharyngeal cancer, and a subset of gastric cancers), contributing to 1.5% of all cancer cases worldwide and approximately 200,000 new cases every year. However, this virus is found in more than 90% of healthy adults worldwide, indicating that EBV infection alone is not enough to cause cancer. The specific geographical distribution of some EBV-associated malignancies (such as Burkitt’s lymphoma in equatorial Africa and nasopharyngeal cancer in East Asia) indicates that the development of an EBV-associated neoplasm requires different environmental and genetic co-factors, of which only some are currently known. 

In this Special Issue, we present a collection of 26 papers (9 research papers and 17 reviews) covering a range of topics related to EBV infection in cancer patients. These fall into three general areas: (1) EBV-encoded genes; (2) EBV and immune responses; and (3) EBV-associated malignancies and EBV-targeted therapies. In this Special Issue, we aim to further elucidate the role of EBV infection in EBV-driven malignancies by reviewing the literature and reporting new findings addressing some of the unanswered questions in the field of EBV.

## 2. EBV-Encoded Genes

EBV is classified into EBV type 1 (EBV-1) and type 2 (EBV-2) based on specific polymorphisms of the EBNA2 and EBNA3A, 3B, and 3C genes. The two types have functional differences in their ability to transform human B cells. EBV-1 can transform B-cells into LCL more efficiently than EBV-2, and EBV-2 preferentially infects T-cell lymphocytes. 

Like other herpesviruses, after primary infection, EBV establishes an asymptomatic, life-long latent infection in a human host, for which occasional reactivation occurs. During latency, EBV expresses a small number of viral genes characterizing distinct latent gene expression patterns; these have been termed 0, I, II, and III. Each latency is dependent on the cell type, the amount of time passed since infection, and the milieu of the cell (e.g., germinal centre or peripheral blood). Upon lytic induction, the expression of the viral immediate early genes BZLF1 and BRLF1 is induced, followed by numerous viral proteins such as BILF1, BMRF1, and BNLF2A. The EBV latency program also involves the expression of non-coding RNAs (ncRNAs), including viral microRNAs. These ncRNAs have different functions that contribute to viral persistence and the development of EBV-associated cancers.

This Special issue includes an original article on EBNA3A and a comprehensive description of BARF1, LMP1, BZLF1, BILF1, and EBV-microRNAs. 

Zanella, L. et al. [1] evaluated more than a thousand EBNA3A sequences of EBV from different human clinical manifestations and geographic locations and found differences in six peptide motifs called nuclear localization signals (NLSs) among the EBV types. EBNA3A from EBV-2 showed that two (NLS3 and NLS4) of the six NLSs were altered (non-canonical) compared with the EBNA3A EBV-1. The authors concluded that these changes could contribute to different cell-type specificities.

Wang, L. et al. [2] summarized the recent research progress regarding LMP1, especially with respect to the novel components LIMD1, p62, and LUBAC in the LMP1 signalosome. They highlighted a new perspective regarding the behaviour and functions of LMP1, which engages different components to manipulate specific cellular processes. In particular, the authors suggested that LMP1 employs (a) p62 to interfere with cellular selective autophagy and antioxidative stress; (b) LIMD1 to repress the anti-oncogenic Hippo pathway; and (c) UCHL1 to regulate the Wnt/β-Catenin/TCF proliferation pathway. 

Lo, A.K.F et al. [3] described the immunomodulatory properties of BARF1 with regard to inhibiting macrophage differentiation and activation. The authors also outlined the contribution of BARF1 to immortalization and malignant transformation through the upregulation of the NF-κB pathway and the induction of genes associated with cell cycle control. Subsequently, the authors discussed the potential strategies for targeting the BARF1 protein and their role as a novel therapy for EBV-driven epithelial cancers.

The work of Germini, D. et al. [4] mainly described BZLF1, which is the principle transcriptional transactivator that switches EBV infection from latency to lytic replication. The authors discussed the structural and functional features of BZLF1 and summarized the currently increasing body of evidence that supports its contribution (direct or indirect) to oncogenesis. Finally, the authors identified BZLF1 as a prognostic or diagnostic marker in lymphomagenesis. 

This Special Issue also includes a review that consolidates the findings of over 15 years of research on BILF1, a G protein-coupled receptor (GPCR), in the context of EBV-specific drug development. The authors have described the contribution of BILF1 to immune evasion, tumorigenesis, and the persistence of EBV^5^. In addition, they summarized the epidemiology of EBV-associated malignancies, the current standard of care, and EBV-specific therapeutics in development [5].

Notarte, K.I. et al. [6] discussed the emerging importance and potential clinical utility of EBV microRNAs and other ncRNAs with respect to Burkitt’s lymphoma (BL), Hodgkin’s lymphoma (HL), diffuse large B cell lymphoma (DLBCL), nasopharyngeal carcinoma (NPC), and gastric cancer (GC).

## 3. EBV and Immune Response

The tumour microenvironment (TME) is a complex and heterogenous ecosystem where neoplastic cells co-exist with immune cells and non-immune cells. The interplay between these elements defines a distinct immunological signature associated with immune responses. Increasing evidence shows that EBV can influence the TME to its own benefit by establishing an immune-suppressive surrounding, thereby facilitating the onset and development of EBV-associated malignancies. 

On this research topic, Forconi, C.S. et al. [7] defined the phenotypes of the EBNA1-specific T cells in BL, one of the most important EBV-associated paediatric tumours. They found that EBNA1-specific T cell responses are highly heterogeneous and are shaped by malaria and EBV-associated immune regulatory adaptations. They also observed an enrichment of IL-10-producing EBNA1-specific T cells and a depletion of IFN-γ+ EBNA1-specific CD4+ T cells, which complementarily contributes to impaired T cell cytotoxicity in eBL pathogenesis.

Stępień, E. et al. [8] studied the expression of Toll-like receptor (TLR) 9, a protein required for congenital immune response to infections with viruses such as EBV, in the serum and tissue of patients with EBV-positive and EBV-negative oropharyngeal squamous cell carcinoma (OPSCC). They showed that in EBV-positive patients, the levels of TLR9 (in serum and tissue) were significantly lower than in EBV-negative patients, whereas no significant relationship was observed between TLR9 levels and IL-10, TNFα, VEGF, and TGFβ levels.

De Fátima Aquino Moreira-Nunes, C. et al. [9] assessed the correlation between EBV status, PD-L1 expression, and overall survival among GC patients. This study revealed that EBV-positive GC presented a high relative expression of PD-L1 and that the high expression of PD-L1 increases the overall survival of GC patients.

Bauer, M. et al. [10] focused on the role of TME in EBV-associated tumours and provided an extensive description of the intrinsic and extrinsic immune evasion strategies adopted by EBV to evade the immune response.

Lino, C.N.R. et al. [11] discussed how inborn mutations in TNFRSF9, CD27, CD70, CORO1A, CTPS1, ITK, MAGT1, RASGRP1, STK4, CARMIL2, SH2D1A, and XIAP affect immunity against EBV, leading to increased susceptibility to lymphoproliferative diseases and lymphomas.

Deng, Y. [12] published an article that examined how EBV-lytic products and co-infections such as Plasmodium falciparum, KSHV, and HIV interact with EBV, modify its immune control, and shape its tumorigenesis. 

## 4. EBV-Associated Malignancies and EBV-Targeted Therapies

Although EBV-associated malignancies have been investigated for over 50 years, virally mediated transformation is not completely understood, and the development of EBV-specific therapeutic strategies is still a major challenge. 

This section comprises (a) nine manuscripts that cover the involvement of EBV in lymphoproliferative disorder (LPD), HL, HIV-related lymphomas, angioimmunoblastic T cell lymphoma (AITL), extra-nodal NK/T cell lymphoma (ENKTCL), NPC, GC, thymic lymphoepithelial carcinoma (TLEC) breast cancer, and laryngeal squamous cell carcinoma (LSSC) and (b) two reviews on novel protective or therapeutic strategies for targeting EBV-associated malignancies.

### 4.1. EBV-Positive B-Cell Lymphoproliferative Disorder

EBV contributes to the reactive and neoplastic lymphoid proliferation of B-, T-, and NK-cell lineages, which represent a vast clinicopathological spectrum ranging from indolent, self-limited disease to aggressive lymphomas. EBV-positive B-cell lymphoproliferative disorder (EBV+ B-LPD) is the most common of these diseases, accounting for 3% to 15% of diffuse large B-cell lymphomas. Ishikawa, E. et al. [13] reviewed EBV+ B-LPD affecting the gastrointestinal tract with a focus on PD-L1 expression in tumour and non-malignant immune cells to better understand this peculiar disease.

### 4.2. Hodgkin’s Lymphoma

Nohtani, M. et al. [14] reviewed the association between EBV and cHL, focusing on the impact of EBV status on clinical outcomes among cHL patients reported in the literature and in a cohort of children and adolescents with cHL from the UK. The authors highlighted the age-related impact of EBV status on outcome in cHL patients and suggested different pathogenic effects of EBV at different stages of life that could be considered in future treatments.

### 4.3. HIV-Related Lymphomas

The incidence of lymphomas is greater among people living with HIV (PLWH). For all HIV-related lymphomas (HRL), the prevalence of Epstein–Barr virus (EBV) is high. Despite the close relationship between EBV and HRL, the impact of EBV on clinical aspects has not been extensively studied. Verdu-Bou, M. et al. [15] reviewed the different EBV-associated lymphomas affecting people with HIV, analysing the influence of EBV on the epidemiology, etiopathogenesis, clinical features, treatment, diagnosis, and prognosis of each lymphoma subtype. 

### 4.4. Angioimmunoblastic T-Cell Lymphoma (AITL)

Angioimmunoblastic T-cell lymphoma (AITL), which is likely the most common peripheral T-cell lymphoma, is very commonly associated with Epstein–Barr virus (EBV), although the virus is not often found in neoplastic T cells but rather in adjacent B cells. Few studies have focused on the EBV–AITL association, and it is not clear what role the virus plays in this pathology. Bayda, N. et al. [16] studied the transcriptome of EBV-positive AITL cases in comparison to other EBV-associated lymphomas and with respect to the results obtained in vitro. They found EBV latency II characterized by the expression of BNLF2a and BCRF1 genes and the highest expression of Bam-HI A rightward transcripts (BARTs) in AITLs, suggesting that these transcripts may participate in the survival of infected cells and the development of AITL. 

### 4.5. NK/T-Cell Lymphoma

Extranodal NK/T-cell lymphoma (ENKTCL) is an aggressive lymphoma associated with EBV infection that occurs mainly in Asian and Latin American populations. In the last decade, the genetic landscape of ENKTCL has been comprehensively characterized using next-generation sequencing (NGS). Montes-Mojarro, I.A. et al. [17] discussed the most important findings regarding EBV pathogenesis and genetics in ENKTCL. The authors also provided a comparison of the EBV strains and LMP1 variants in different populations and highlighted the main therapeutically targetable pathways in ENKTCL oncogenesis (including the JAK–STAT signalling pathway, immune response evasion, MYC overexpression, and epigenetic alterations).

### 4.6. Nasopharyngeal Carcinoma (NPC)

Nasopharyngeal carcinoma (NPC) is one of the most common tumours in China and Southeast Asia. The aetiology of NPC seems to be complex and involves many determinants, one of which is EBV infection. However, the exact relationship between EBV and in NPC is unknown. 

Yu, F. et al. [18] elucidated the mechanisms by which EBV transforms the nasopharyngeal epithelium, providing the first-ever in vitro model of primary nasopharyngeal epithelial cells that developed pseudostratified epithelia and were susceptible to EBV infection. The authors observed that EBV infection disrupted the integrity of the epithelium, while cell differentiation promoted lytic viral replication. This infection model constitutes a significant contribution to this field, enabling researchers to investigate the pathogenesis of pre-neoplastic EBV-infected cells and develop anti-EBV chemotherapeutic agents to treat NPC patients.

Emanuel, O. et al. [19] offered a comprehensive and up-to-date summary of NPC, focusing on the role of somatostatin receptor 2 (SSTR2), a key tumour biomarker and promising target for imaging and therapy.

Richardo, T. et al. [20] provided a comprehensive overview covering recent insights into the pathogenic role of EBV infection in NPC. The authors focused on cancer hallmarks associated with EBV in NPC development and highlighted the cellular signalling pathways (including Wnt/β-catenin, JAK/STAT, PI3k/Akt/mTOR, EGFR/MAPK, and NF-κB pathways) that are modulated by EBV oncoproteins or non-coding RNAs.

### 4.7. Gastric Cancer (GC)

EBV-associated gastric cancer (GC) is one of four major gastric cancer types and is traditionally considered to be related to lymphoepithelioma-like GC. Few studies have investigated the clinical significance of EBV infection in intestinal/solid-type, diffuse (poorly cohesive)-type, and lymphoepithelioma-like GC. Fang, W.L. et al. [21] analysed the clinicopathological features, genetic alterations, and prognoses of a total of 460 GC patients with and without EBV infection that were receiving curative surgery. The authors showed that the intestinal/solid-type GC patients with EBV-positive tumours were associated with higher PD-L1 expression and more liver metastases, while the lymphoepithelioma-like GC patients with EBV-positive tumours presented more PI3K/AKT pathway mutations.

### 4.8. Thymic Lymphoepithelial Carcinoma (TLEC)

Thymic lymphoepithelial carcinoma (TLEC) is a rare primary thymic carcinoma. Though there have been reports of thymic carcinoma, including TLEC, there are few studies on the analysis of TLEC alone, and only case reports have been stated. In this Special Issue, Ose, N. et al. [22] studied 58 cases from 34 reports and concluded that the poor prognosis of this malignancy is not considered to be affected by the presence or absence of EBV positivity but rather by the advanced stage at which TLEC is detected.

### 4.9. Breast Cancer (BC)

While the relationship between EBV infection and several types of human lymphomas (e.g., BL and HL) as well as other epithelial cancers (e.g., gastric cancer and NPC) has been clearly established, recent investigations have revealed the possible involvement of EBV in breast cancer (BC). To date, the link between EBV and this tumour is still controversial. Sinclair, A.J. et al. [23] reviewed and discussed the evidence for and against the association between EBV and breast cancer and posed questions that could help answer whether EBV is a cause of this tumour. The authors concluded that the evidence for the presence of EBV in BC biopsies is concentrated in specific geographic regions but that its breadth is currently insufficient for providing a causal link. 

### 4.10. Laryngeal Squamous Cell Carcinoma (LSCC)

Increasing molecular evidence supports EBV’s involvement in the pathogenesis of laryngeal squamous cell carcinoma (LSCC); however, the epidemiological data are inconsistent. In their retrospective case-control study, Lee, L.A. et al. [24] aimed to determine whether EBV infection underlies the risk and prognosis of LSCC. The authors identified EBV DNA positivity, older age, cigarette smoking, and higher expression of BCL-2, B2M, and CD161 as risk factors for primary LSCC, whereas high EBER signals and low CD3 expression behaviour were identified as independent predictors of local recurrence and disease-free survival within five years. The information obtained in this work may guide future prevention, treatment, and follow-up strategies for the study of EBV infections in cases of laryngeal cancer. 

This Special Issue also includes two manuscripts that discuss the new EBV-targeted therapies currently under development for EBV-associated malignancies. Pei, Y. et al. [25] described small-molecule inhibitors, immunotherapy, cell therapy, preventative and therapeutic vaccines, and other potent approaches that constitute novel strategies for preventing and treating EBV-lymphomas. Yiu, S.P.T. et al. [26] discussed the novel EBV lytic induction therapy, which is an emerging virus-targeted therapeutic approach that exploits the presence of EBV in tumour cells to confer specific killing effects against the virus. The authors also reviewed the current knowledge on EBV lytic reactivation, the major weaknesses of translating lytic induction therapy into clinical settings, and some potential strategies for the future development of this therapy for EBV-related lymphoid and epithelial malignancies.

## References

[1] Zanella L., Reyes M.E., Riquelme I., Abanto M., León D., Viscarra T., Ili C., Brebi P. (2021). Genetic Patterns Found in the Nuclear Localization Signals (NLSs) Associated with EBV-1 and EBV-2 Provide New Insights into Their Contribution to Different Cell-Type Specificities. Cancers.

[2] Wang L., Ning S. (2021). New Look of EBV LMP1 Signaling Landscape. Cancers.

[3] Lo A.K.-F., Dawson C.W., Lung H.L., Wong K.-L., Young L.S. (2020). The Therapeutic Potential of Targeting BARF1 in EBV-Associated Malignancies. Cancers.

[4] Germini D., Sall F., Shmakova A., Wiels J., Dokudovskaya S., Drouet E., Vassetzky Y. (2020). Oncogenic Properties of the EBV ZEBRA Protein. Cancers.

[5] Knerr J.M., Kledal T.N., Rosenkilde M.M. (2021). Molecular Properties and Therapeutic Targeting of the EBV-Encoded Receptor BILF1. Cancers.

[6] Notarte K.I., Senanayake S., Macaranas I., Albano P.M., Mundo L., Fennell E., Leoncini L., Murray P. (2021). MicroRNA and Other Non-Coding RNAs in Epstein–Barr Virus-Associated Cancers. Cancers.

[7] Forconi C.S., Mulama D.H., Saikumar Lakshmi P., Foley J., Otieno J.A., Kurtis J.D., Berg L.J., Ong’echa J.M., Münz C., Motorman A.M. (2021). Interplay between IL-10, IFN-γ, IL-17A and PD-1 Expressing EBNA1-Specific CD4+ and CD8+ T Cell Responses in the Etiologic Pathway to Endemic Burkitt Lymphoma. Cancers.

[8] Stępień E., Strycharz-Dudziak M., Malm M., Drop B., Polz-Dacewicz M. (2021). Serum and Tissue Level of TLR9 in EBV-Associated Oropharyngeal Cancer. Cancers.

[9] De Fátima Aquino Moreira-Nunes C., de Souza Almeida Titan Martins C.N., Feio D., Lima I.K., Lamarão L.M., de Souza C.R.T., Costa I.B., da Silva Maués J.H., Soares P.C., de Assumpção P.P. (2021). PD-L1 Expression Associated with Epstein—Barr Virus Status and Patients’ Survival in a Large Cohort of Gastric Cancer Patients in Northern Brazil. Cancers.

[10] Bauer M., Jasinski-Bergner S., Mandelboim O., Wickenhauser C., Seliger B. (2021). Epstein–Barr Virus—Associated Malignancies and Immune Escape: The Role of the Tumor Microenvironment and Tumor Cell Evasion Strategies. Cancers.

[11] Lino C.N.R., Ghosh S. (2021). Epstein–Barr Virus in Inborn Immunodeficiency—More Than Infection. Cancers.

[12] Deng Y., Münz C. (2021). Roles of Lytic Viral Replication and Co-Infections in the Oncogenesis and Immune Control of the Epstein–Barr Virus. Cancers.

[13] Ishikawa E., Satou A., Nakamura M., Nakamura S., Fujishiro M. (2021). Epstein-Barr Virus Positive B-Cell Lymphoproliferative Disorder of the Gastrointestinal Tract. Cancers.

[14] Nohtani M., Vrzalikova K., Ibrahim M., Powell J.E., Fennell É., Morgan S., Grundy R., McCarthy K., Dewberry S., Bouchal J. (2022). Impact of Tumour Epstein–Barr Virus Status on Clinical Outcome in Patients with Classical Hodgkin Lymphoma (cHL): A Review of the Literature and Analysis of a Clinical Trial Cohort of Children with cHL. Cancers.

[15] Verdu-Bou M., Tapia G., Hernandez-Rodriguez A., Navarro J.-T. (2021). Clinical and Therapeutic Implications of Epstein–Barr Virus in HIV-Related Lymphomas. Cancers.

[16] Bayda N., Tilloy V., Chaunavel A., Bahri R., Halabi M.A., Feuillard J., Jaccard A., Ranger-Rogez S. (2021). Comprehensive Epstein-Barr Virus Transcriptome by RNA-Sequencing in Angioimmunoblastic T Cell Lymphoma (AITL) and Other Lymphomas. Cancers.

[17] Montes-Mojarro I.A., Fend F., Quintanilla-Martinez L. (2021). EBV and the Pathogenesis of NK/T Cell Lymphoma. Cancers.

[18] Yu F., Lu Y., Li Y., Uchio Y., Pangnguriseng U.A., Kartika A.V., Iizasa H., Yoshiyama H., Loh K.S. (2021). Epstein–Barr Virus Infection of Pseudostratified Nasopharyngeal Epithelium Disrupts Epithelial Integrity. Cancers.

[19] Emanuel O., Liu J., Schartinger V.H., Nei W.L., Chan Y.Y., Tsang C.M., Riechelmann H., Masterson L., Haybaeck J., Oppermann U. (2021). SSTR2 in Nasopharyngeal Carcinoma: Relationship with Latent EBV Infection and Potential as a Therapeutic Target. Cancers.

[20] Richardo T., Prattapong P., Ngernsombat C., Wisetyaningsih N., Iizasa H., Yoshiyama H., Janvilisri T. (2020). Epstein-Barr Virus Mediated Signaling in Nasopharyngeal Carcinoma Carcinogenesis. Cancers.

[21] Fang W.-L., Chen M.-H., Huang K.-H., Lin C.-H., Chao Y., Lo S.-S., Li A.F.-Y., Wu C.-W., Shyr Y.-M. (2020). The Clinicopathological Features and Genetic Alterations in Epstein–Barr Virus-Associated Gastric Cancer Patients after Curative Surgery. Cancers.

[22] Ose N., Kawagishi S., Funaki S., Kanou T., Fukui E., Kimura K., Minami M., Shintani Y. (2021). Thymic Lymphoepithelial Carcinoma Associated with Epstein-Barr Virus: Experiences and Literature Review. Cancers.

[23] Sinclair A.J., Moalwi M.H., Amoaten T. (2021). Is EBV Associated with Breast Cancer in Specific Geographic Locations?. Cancers.

[24] Lee L.-A., Fang T.-J., Li H.-Y., Chuang H.-H., Kang C.-J., Chang K.-P., Liao C.-T., Chen T.-C., Huang C.-G., Yen T.-C. (2021). Effects of Epstein-Barr Virus Infection on the Risk and Prognosis of Primary Laryngeal Squamous Cell Carcinoma: A Hospital-Based Case-Control Study in Taiwan. Cancers.

[25] Pei Y., Wong J.H.Y., Robertson E.S. (2020). Targeted Therapies for Epstein-Barr Virus-Associated Lymphomas. Cancers.

[26] Yiu S.P.T., Dorothea M., Hui K.F., Chiang A.K.S. (2020). Lytic Induction Therapy against Epstein–Barr Virus-Associated Malignancies: Past, Present, and Future. Cancers.

